# Editorial: Whole-Body Electromyostimulation: A Training Technology to Improve Health and Performance in Humans?

**DOI:** 10.3389/fphys.2020.00523

**Published:** 2020-05-26

**Authors:** Wolfgang Kemmler, Heinz Kleinöder, Michael Fröhlich

**Affiliations:** ^1^Institute of Medical Physics, Friedrich-Alexander University of Erlangen-Nürnberg, Erlangen, Germany; ^2^Institute of Training Science and Sport Informatics, German Sport University Cologne, Cologne, Germany; ^3^Department of Sports Science, Technische Universität Kaiserslautern, Kaiserslautern, Germany

**Keywords:** whole-body electromyostimulation, exercise, health, performance, training technology

Originally created and commercially launched in Germany in 2009, whole-body electromyostimulation (WB-EMS) is a promising training technology with rapid and widespread dissemination particularly in Europe and the Far East. Even though there are more than 2,000 commercial WB-EMS providers with about 250,000 clients in Germany alone, research on WB-EMS is still limited. Symptomatically, there is not even a mandatory definition of WB-EMS. Thus, we would suggest defining WB-EMS as “simultaneous application of electric stimuli via at least six current channels or participation of all major muscle groups, with a current impulse effective to trigger muscular adaptations.” This concurrent stimulation of large muscle areas, each with dedicated impulse intensity, delivers the “time effectiveness” of WB-EMS, a key feature of this training method. However, apart from its “efficiency,” EMS technology applied locally or globally enables a supramaximal workload without high voluntary effort (Paillard; Watanabe et al.). This unique feature of efficiency and high workload with low voluntary effort may explain the steadily growing attractiveness of WB-EMS for health, fitness, and performance professionals. The present Research Topic on WB-EMS thus aimed to stimulate incentivize dedicated research in all these disciplines.

Since completion of the present Research Topic, 15 of 24 submitted articles have been accepted. Simplified, five contributions (Berger et al.; Ludwig et al.; Paillard; Watanabe et al.; Zart et al.) focus on basic EMS research, predominately to derive optimized WB-EMS protocols. While the German research group (Berger et al.; Ludwig et al.; Zart et al.) focused on dedicated strain parameters, Watanabe et al. and Paillard addressed the interaction of WB-EMS and voluntary contraction in humans. Apart from other important findings highly relevant for practical application, one key message can be derived from both basic research and the performance studies listed below: Evidence suggests that simultaneously applied WB-EMS did not increase the effects of maximum voluntary contractions. Thus, when exercising with WB-EMS, impulse parameters and not high voluntary effort are the decisive effectors.

With seven studies, the majority of projects address the fitness and performance domain. In summary, the trials included recreational runners (Amaro-Gahete et al.; Amaro-Gahete et al.), sports students (Dörmann et al.; Wirtz et al.), amateur ice-hockey players (Schuhbeck et al.), and professional soccer players (Filipovic, DeMarees et al.; Filipovic, Bizjak et al.). Apart from functional outcomes e.g., sprint and jump performance, shoot speed, strength, and power (Amaro-Gahete et al.; Amaro-Gahete et al.; Dörmann et al.; Filipovic, DeMarees et al.; Schuhbeck et al.; Wirtz et al.) amenable to resistance-type WB-EMS, Amaro-Gahete et al. and Amaro-Gahete et al. reported WB-EMS induced improvements in running performance after volume reductions in recreational runners. This finding on endurance capacity was not confirmed by Filipovic, Bizjak et al., however, who found no relevant WB-EMS effects on VO_2_max and various blood parameters related to oxygen supply in professional soccer players.

Reviewing the present literature on WB-EMS, with only three contributions that addressed health related issues (Schink et al.; Willert et al.; Teschler and Mooren), the “WB-EMS and health” domain was considerably underrepresented in our Research Topic. While Teschler and Mooren reviewed negative side-effects of WB-EMS, a topic that will be addressed in more depth later, two research groups from Erlangen, Germany (Schink et al.; Willert et al.) addressed interactions of WB-EMS and dietary supplements. While Schink et al. determined the effect of combined WB-EMS and dietary support on body composition, physical function, quality of life, and blood parameters in patients with hematologic malignancies, Willert et al. evaluated effects of WB-EMS and protein supplementation on energy-restriction-induced loss of muscle mass during intended weight reduction.

Although it is far from clear which composition of exercise and/or impulse parameters might be optimum or even appropriate for a given outcome (Berger et al.; Ludwig et al.; Paillard; Watanabe et al.; Zart et al.), in essence most of the studies indicated that WB-EMS can be indeed be considered an effective training technology for improving health- and performance-related parameters. Transferred into clinical practice, WB-EMS might thus be an option for people with low time resources and inability or unwillingness to exercise conventionally. This also includes athletes looking for time-efficient exercise protocols to impact secondary training aims related to strength and power.

However, beside effectiveness, other less positive aspects of WB-EMS fall short in the present Research Topic. Based on its artificial application, physiological mechanisms that protect against overloading during conventional training do not come into play during EMS. Thus, considering that even local EMS application might induce severe rhabdomyolysis (Johannsen and Krogh, 2019), it is obvious that a technology able to stimulate up to 2,600 cm^2^ of muscular area simultaneously entails a high risk of triggering unintended effects (Teschler and Mooren)—at least when inappropriately applied (Kemmler et al., 2016). Indeed, severe rhabdomyolysis was frequently reported (e.g., Stollberger and Finsterer, 2019) in particular after improper i.e., too intense, first WB-EMS sessions. Of note, these negative side effects led to a temporary ban of WB-EMS in Israel in 2015. As a result many researchers call for stronger regulation of WB-EMS (e.g., Malnick et al., 2016). In Germany, several recommendations for safe and effective WB-EMS (Kemmler et al., 2016, 2019) have been launched. In 2018, DIN (German Industry Norm) 33961-5, a German standard regulating the application of WB-EMS in commercial, non-medical settings in depth, was released. In parallel, the German “Federal Ministry for the Environment, Nature Conservation, and Nuclear Safety (BMU)” published the revised German Radiation Protection Statutes, a mandatory guideline that includes WB-EMS (“applications of non-ionizing radiation to humans”; NISV) (BMU, 2019). The NISV cover in particular aspects of operation, information, documentation, and the mandatory requirements for qualification as EMS trainer. However, apart from the latter aspect, we are not convinced that the formal requirements specified by the NiSV will contribute to fewer adverse effects and increased effectiveness of WB-EMS. Thus, we would like to take the opportunity to clearly state our position on aspects of WB-EMS application under ongoing discussion.

Firstly, we strongly support DIN 33961-5 (Kemmler et al., 2019) with its relative and absolute contraindications for commercial, non-medical WB-EMS application. Nevertheless, we are aware of the problem that people falling within the absolute or relative contraindications have been excluded from or not allowed to apply WB-EMS due to their physicians' unjustified concerns, although WB-EMS might be the most suitable training option for them. Generating scientific evidence might lead to a revision of the contraindications in the nearest future, allowing more people to use this time-efficient, joint friendly and tailored exercise intervention. Further, as mentioned, DIN 33961-5 covers commercial, non-medical providers; medical providers with an even more individualized WB-EMS application under medical supervision were not addressed. However, as physicians will still act as gatekeepers for questionable WB-EMS application, and considering their crucial role during instructor education (BMU, 2019), at least training programs for sports medicine and physical rehabilitation should include aspects of WB-EMS application.

Second, we consider very close support by attentive, well-trained, and mandatorily licensed instructors as the key factor of safe and effective WB-EMS application. The close interaction and narrow distance between instructor and participant necessary to ensure especially (1) frequent feedback from the participant about perceived exertion for each area of stimulation, (2) permanent visual monitoring of the participant and eye contact to check participant strain, avoid overload, and to react immediately to the first signs of cardiorespiratory or metabolic side effect, and (3) verbal and haptic movement corrections and rapid assistance in cases of emergency, particularly cutting off power supply of the device, leads us to strongly advise a 1:1 instructor-participant ratio; although a 1:2 ratio is also considered tolerable for non-medical WB-EMS application with less critical participants.

We feel that this Research Topic provides additional evidence for the health, fitness and performance aspects WB-EMS application. We are aware that a plethora of research questions with respect to the most optimum WB-EMS protocol for given outcomes and varying target populations remained to be addressed. However, we conclude that some key aspects of WB-EMS should be addressed with particular emphasis in the nearest future. From a sport scientific point of view, intensity regulation by objective strain parameters based on advanced biomarkers might further increase the safety and effectiveness of WB-EMS. In parallel, the evaluation of progression models is essential for ensuring the sustainability of WB-EMS effects. To date, only few studies exceed a period of 6 months, thus longer trials have to monitor WB-EMS effectiveness and safety. Considering the time effectiveness, joint friendliness, low voluntary effort and customization of WB-EMS; from a health perspective, further research should particularly focus on diseases (e.g., multiple sclerosis, diabetes mellitus, selected types of cancer, hypertonia, arthritis) with limited potential or perspective for conventional exercise. The permissive, additive or synergistic effect of other low-threshold interventions (e.g., dietary supplements) combined with WB-EMS should also be addressed more forcefully. However, apart from health issues, the feasibility and effectiveness in settings with low time and spatial resources but high physical demands (e.g., Naval and Special Forces, Fire Fighters) will be challenging.

## Author Contributions

All authors listed have made a substantial, direct and intellectual contribution to the work, and approved it for publication.

## Conflict of Interest

The authors declare that the research was conducted in the absence of any commercial or financial relationships that could be construed as a potential conflict of interest.
